# Cardiovascular risk estimation in women with a history of hypertensive pregnancy disorders at term: a longitudinal follow-up study

**DOI:** 10.1186/1471-2393-13-126

**Published:** 2013-06-04

**Authors:** Wietske Hermes, Jouke T Tamsma, Diana C Grootendorst, Arie Franx, Joris van der Post, Maria G van Pampus, Kitty WM Bloemenkamp, Martina Porath, Ben W Mol, Christianne JM de Groot

**Affiliations:** 1 Department of Obstetrics and Gynecology, Medical Center Haaglanden, The Hague, the Netherlands; 2 Department of Obstetrics, VU University Medical Center, Amsterdam, the Netherlands; 3 Department of Internal Medicine, Leiden University Medical Center, Leiden, the Netherlands; 4 Department of Clinical Epidemiology, Landsteiner Institute, Medical Center Haaglanden, The Hague, the Netherlands; 5 Division of Woman and Baby, University Medical Center, Utrecht, the Netherlands; 6 Department of Obstetrics and Gynecology, Amsterdam Medical Center, Amsterdam, the Netherlands; 7 Department of Obstetrics and Gynecology, Onze Lieve Vrouwe Gasthuis, Amsterdam, the Netherlands; 8 Department of Obstetrics, Leiden University Medical Center, Leiden, the Netherlands; 9 Department of Obstetrics and Gynecology, Maxima Medical Center, Veldhoven, the Netherlands

**Keywords:** Cardiovascular risk, Cardiovascular risk prediction, Follow-up study, Gestational hypertension, Preeclampsia

## Abstract

**Background:**

Cardiovascular disease is associated with major morbidity and mortality in women in the Western world. Prediction of an individual cardiovascular disease risk in young women is difficult. It is known that women with hypertensive pregnancy complications have an increased risk for developing cardiovascular disease in later life and pregnancy might be used as a cardiovascular stress test to identify women who are at high risk for cardiovascular disease. In this study we assess the possibility of long term cardiovascular risk prediction in women with a history of hypertensive pregnancy disorders at term.

**Methods:**

In a longitudinal follow-up study, between June 2008 and November 2010, 300 women with a history of hypertensive pregnancy disorders at term (HTP cohort) and 94 women with a history of normotensive pregnancies at term (NTP cohort) were included. From the cardiovascular risk status that was known two years after index pregnancy we calculated individual (extrapolated) 10-and 30-year cardiovascular event risks using four different risk prediction models including the Framingham risk score, the SCORE score and the Reynolds risk score. Continuous data were analyzed using the Student’s T test and Mann–Whitney U test and categorical data by the Chi-squared test. A poisson regression analysis was performed to calculate the incidence risk ratios and corresponding 95% confidence intervals for the different cardiovascular risk estimation categories.

**Results:**

After a mean follow-up of 2.5 years, HTP women had significantly higher mean (SD) extrapolated 10-year cardiovascular event risks (HTP 7.2% (3.7); NTP 4.4% (1.9) (p<.001, IRR 5.8, 95% CI 1.9 to 19)) and 30-year cardiovascular event risks (HTP 11% (7.6); NTP 7.3% (3.5) (p<.001, IRR 2.7, 95% CI 1.6 to 4.5)) as compared to NTP women calculated by the Framingham risk scores. The SCORE score and the Reynolds risk score showed similar significant results.

**Conclusions:**

Women with a history of gestational hypertension or preeclampsia at term have higher predicted (extrapolated) 10-year and 30-year cardiovascular event risks as compared to women with a history of uncomplicated pregnancies. Further large prospective studies have to evaluate whether hypertensive pregnancy disorders have to be included as an independent variable in cardiovascular risk prediction models for women.

## Background

Cardiovascular disease is associated with major morbidity and mortality in women in the Western world [1]. Women are less likely to receive appropriate cardiovascular preventive care compared to men and heart disease in women is not always recognized as a major health care concern [2]. Several epidemiological studies have demonstrated the association between hypertensive pregnancy disorders and cardiovascular morbidity and mortality in later life [3-9]. Subsequently, it has been suggested that pregnancy may act as an early natural “*stress test*” unmasking underlying defects and thereby identifying women at high risk for cardiovascular disease in later life [10].

Worldwide, different risk prediction models have been developed for individual risk prediction of cardiovascular disease in both apparently healthy men and women [11-17]. Currently, the Framingham risk score is the most compared score in literature and widely used in North American countries [18], while the systematic coronary risk evaluation (SCORE) score has been advised by the European guidelines [19]. In spite of the existence and use of several risk prediction models, it remains a great challenge to determine which *specific* woman is at high risk for cardiovascular disease, especially in *young* women.

Obstetric history, e.g. hypertensive pregnancy disorders, is not included as a variable in cardiovascular risk prediction models. However, obstetric history may help to identify women, who are at high risk for cardiovascular disease in later life; they may benefit from early cardiovascular risk screening after their complicated pregnancy together with subsequent individual cardiovascular risk prediction and primary prevention programs [20,21]. Mosca et al. showed that women, who perceive themselves at risk for cardiovascular disease and know the goals for prevention, are motivated to take action toward a heart healthy life style [2,22].

In the present study, we assess cardiovascular event risks in women with a history of gestational hypertension or preeclampsia at term using four previous described validated cardiovascular risk prediction models, including the 10-year Framingham risk score, the 30-year Framingham risk score, the 10-year estimation by the SCORE score, and the 10-year estimation by the Reynolds risk score. According to the European cardiovascular risk factor management guidelines for young women with elevated risk factor levels [23], we extrapolate the 10-year cardiovascular disease risks as if the woman is 60 years old. The aim of this study is to compare these estimated (extrapolated) 10-year and 30-year cardiovascular disease risks between women with a history of term gestational hypertension or term preeclampsia and women with a history of term normotensive pregnancies, in order to improve assessment of reliable cardiovascular risk estimation for the long term cardiovascular disease outcome in relatively young women.

## Methods

### Participants

We studied 300 women who had delivered in the Netherlands between 2005 and 2008, with the diagnosis term gestational hypertension or term preeclampsia after 36 weeks' gestation and 94 healthy control women after uncomplicated term pregnancies. The women were invited for participation in the current follow-up study on cardiovascular event risk estimation.

We enrolled women with a history of term gestational hypertension or term preeclampsia from the Hypertension and Pre-eclampsia Intervention Trial At Term (the HYPITAT study) [24]. Control women were friends of the patients or women from midwifery practices from three different locations in the Netherlands (Groningen, Leiden and The Hague) and they were required to have only previous uncomplicated normotensive pregnancies. A detailed description of all inclusion and exclusion criteria was previously published elsewhere [25,26] Exclusion criteria for participation in the HYPITAT trial and the follow-up study included: antihypertensive medication use for chronic hypertension, diabetes mellitus, gestational diabetes treated with insulin, renal disease, heart disease, previous caesarean section, hemolysis elevated liver enzymes and low platelets syndrome (HELLP), oliguria of less than 500 mL per 24 hours, pulmonary edema or cyanosis, human immunodeficiency virus (HIV), use of intravenous antihypertensive medication, fetal anomalies, intrauterine growth restriction (IUGR), and abnormal fetal- heart rate monitoring. Exclusion criteria for the NTP cohort included HELLP, gestational hypertension, preeclampsia, pre-existing hypertension, (gestational) diabetes, premature delivery, delivery of a neonate with intra uterine growth restriction (below the 5^th^ percentile), renal disease, heart disease, and HIV.

Pregnant and lactating women (within the last three months) were excluded from the follow-up study.

The study was approved by the Institutional Review Board of the University of Leiden (HYPITAT: P04.210) and locally approved by the hospital board of the participating hospitals.

### Definitions

Gestational hypertension was defined as diastolic blood pressure of 95 mmHg or higher measured on two occasions at least six hours apart. Preeclampsia was defined as diastolic blood pressure of 90 mmHg or higher measured on two occasions at least six hours apart, combined with proteinuria (two or more occurrences of protein on a dipstick, > 300 mg total protein within a 24 hours urine collection, or ratio of protein to creatinine > 30 mg/mmol). Severe gestational hypertension or severe preeclampsia were defined as either systolic blood pressure of 170 mmHg or higher, diastolic blood pressure of 110 mmHg or higher, or proteinuria of 5 gram or higher per 24 hours.

Hypertension at follow-up was defined as systolic blood pressure ≥ 140 mmHg, or a diastolic blood pressure ≥ 90 mmHg or current use of antihypertensive medication.

### Classic cardiovascular risk factor assessment

A detailed description of the cardiovascular risk factor assessment and laboratory procedures has been published elsewhere [25]. In short, after enrolment all participants were invited for cardiovascular risk factor assessment. After written informed consent they were asked to complete a questionnaire including questions about their medical history, current medication use, obstetric history, subsequent pregnancy after index pregnancy and family history, including cardiovascular disease. Cardiovascular risk factor assessment included: blood pressure measurement, height and weight (with calculated body mass index (BMI)), and fasting venous blood sample drawing, assayed for: glucose, HDL cholesterol, triglycerides and (high sensitive) C-reactive protein.

### Individual cardiovascular risk prediction

For the prediction of the 10-year general cardiovascular disease risk by the Framingham risk score, cardiovascular risk factors were used according to the methodology reported by D’Agostino et al. [27], i.e. age, total cholesterol, HDL cholesterol, systolic blood pressure, treatment for hypertension, and smoking. 10-Year general cardiovascular disease was defined as coronary death, myocardial infarction, coronary insufficiency, angina, ischemic stroke, hemorrhagic stroke, transient ischemic attack, peripheral artery disease, and heart failure.

30-Year full cardiovascular disease estimation by the Framingham risk score was calculated using the algorithm reported by Pencina et al. [15] 30-Year full cardiovascular disease was defined as coronary death, myocardial infarction, fatal and non-fatal stroke, coronary insufficiency, angina pectoris, transient ischemic attack, intermittent claudication and congestive heart failure.

For prediction of 10-year risk of fatal cardiovascular disease by the SCORE score estimation, risk factors were used according to the algorithm reported by Conroy et al. [11], including age, total cholesterol, systolic blood pressure and smoking. Fatal cardiovascular disease was defined as ICD-9 codes 798.1, 798.2, 401 through 414 and 426 through 443, with the exception of the ICD-9 codes: 426.7, 429.0, 430.0, 432.1, 437.3, 437.4 and 437.5.

For prediction of global cardiovascular disease risk by the Reynolds risk score we used cardiovascular risk factors according to the algorithm reported by Ridker et al., including systolic blood pressure, smoking, total cholesterol, HDL cholesterol, hsCRP and a family history of myocardial infarction < 60 years in first- degree relative [16]. They defined global cardiovascular disease as incident myocardial infarction, stroke, coronary revascularization, or cardiovascular death.

### Sample size and statistical analysis

Our power analysis was based on cardiovascular risk estimation based on the Framingham Heart Study [17]. Due to the young age of our participants, the estimated absolute 10-year cardiovascular risk was likely to be low. Therefore, our approach was to estimate the risk for each woman as if the woman was 60 years old. This approach has been recommended in the cardiovascular risk factor management guidelines for young women with elevated risk factor levels [23]. For detecting an estimated absolute 10-year cardiovascular risk difference between women with a history of term gestational hypertension or term preeclampsia (HTP) and women with a history of normotensive pregnancies (NTP) of 10% increase after extrapolating to the age of 60, we needed a sample size of 456 women for 80% power and a 5% type 1 error probability (two sided), for inclusion in 3:1 ratio (3 HTP: 1 NTP). According to earlier studies [28-30] we expected a homogeneous effect with low prevalence of unfavorable cardiovascular risk factors in normotensive term pregnancy women. Therefore, we have used a 3:1 (HTP:NTP) inclusion ratio instead of 1:1 as we assumed that including more normotensive pregnancy women in 1:1 ratio would have no additional value in this study as a result of homogeneous outcome of cardiovascular risks.

Data were analyzed using SPSS software (version 20.0). Baseline continuous data were expressed as means and standard deviations or as medians and interquartile ranges for not normally distributed values; dichotomous data were presented as numbers and percentages. Comparison of continuous data with a skewed distribution was performed using the non- parametric Mann–Whitney U test and categorical data by the Chi-squared test. A poisson regression analysis was performed to calculate the incidence risk ratios and corresponding 95% confidence intervals for the different cardiovascular risk estimation categories. We made adjustments for potential confounders, where appropriate, including parity (continuous variable) and current BMI (continuous variable). For all tests, a p-value < 0.05 indicated statistical significance.

## Results

Between June 2008 and November 2010, a total of 300 women with a history of term gestational hypertension or term preeclampsia and 94 women with a history of normotensive term pregnancies were included in this follow-up study. Of the eligible 751 women with a history of term gestational hypertension or term preeclampsia, 168 women declined participation in the follow-up study, 6 women refused blood drawing, 175 women were lost to follow-up, 101 women were pregnant or lactating and 1 woman had died in a car accident.

At index pregnancy, women with term gestational hypertension or term preeclampsia were more often nulliparous, had higher body mass index at the first antenatal visit, higher systolic and diastolic blood pressures at the first antenatal visit, lower gestational age at delivery and lower birth weight compared with women with a history of normotensive term pregnancies (Table 1). At 2.5 year follow-up women with a history of term gestational hypertension or term preeclampsia were more often primiparous, used more antihypertensive medication, and had higher body mass index, higher systolic and diastolic blood pressures and higher waist circumferences compared with women with a history of normotensive term pregnancy. There were no significant differences in age, elapsed time since delivery and smoking rates (Table 2).

**Table 1:** Baseline characteristics at index pregnancy^*^

**Characteristics**	**HTP cohort**	**NTP cohort**	**p value**
	**(n=300)**	**(n=94)**	
Maternal age at delivery (years)	31 (28–35)	31 (28–34)	.82
Ethnic origin: Caucasian	273 (89%)	94 (95%)	.14
Other	30 (10%)	5 (5%)	
Unknown	3 (1%)	0 (0%)	
Nulliparous	211 (69%)	30 (30%)	< .001
Systolic blood pressure at first antenatal visit (mmHg)^‡^	120 (110–130)	110 (109–120)	<.001
Diastolic blood pressure at first antenatal visit (mmHg)^‡^	75 (68–80)	65 (60–70)	<.001
BMI at first antenatal visit (kg/m^2^)	25.2 (22.5-29.1)	22.7 (21.2-24.3)	<.001
Gestational age at delivery (weeks)	39.5 (38.4-40.2)	39.9 (39.3-40.7)	.001
Birth weight (gram)	3398 (3030–3710)	3693 (3216–3942)	<.001

^*^Continuous data are expressed as median (IQR), dichotomous data as number of patients (%).

*HTP* women with a history of term gestational hypertension or term preeclampsia.

*NTP* women with a history of normotensive term pregnancies.

^‡ =^ 38 HTP women (12%) had a systolic blood pressure of ≥ 140 mmHg or a diastolic blood pressure of ≥ 90 mmHg at the first antenatal visit (without use of antihypertensive medication).

**Table 2:** **Outcome characteristics of the follow**-**up study**, **2**.5 years postpartum*

**Characteristics**	**HTP cohort**	**NTP cohort**	**p value**
	**(n=300)**	**(n=94)**	
Age at follow up (years)	34 (30–37)	34 (31–37)	.80
Time elapsed since delivery (years)	2.4 (2.2-2.7)	2.5 (2.2-2.9)	.54
Primiparous	134 (44%)	21 (22%)	< .001
Smoking	60 (20%)	19 (19%)	.82
BMI at follow up (kg/m^2^)	26.6 (23.8-30.5)	23.2 (21.8-25.5)	<.001
Antihypertensive medication use	29 (10%)	0 (0%)	.001
Hypertension	104 (35%)	1 (1%)	<.001
Systolic blood pressure (mmHg)	124 (115–130)	110 (105–118)	<.001
Diastolic blood pressure (mmHg)	80 (78–90)	74 (66–80)	<.001
Family history of MI^**^ < 60 years in first- degree relative	48 (16%)	11 (11%)	.21

^*^Continuous data are expressed as median (IQR), dichotomous data as number of patients (%).

^**^MI Myocardial infarction.

*HTP* women with a history of term gestational hypertension or term preeclampsia.

*NTP* women with a history of normotensive term pregnancies.

Biochemical cardiovascular risk factors 2.5 years postpartum, including glucose, HsCRP, total cholesterol, HDL cholesterol and triglycerides, were all significantly higher in women with a history of term gestational hypertension or term preeclampsia compared with women with a history of normotensive term pregnancies (Table 3).

**Table 3:** **Cardiovascular biomarkers in HTP women and NTP women 2**.5 years postpartum^*^

**Cardiovascular biomarker**	**HTP cohort**	**NTP cohort**	***p *value**
	**(n=300)**	**(n=94)**	
Glucose, mg/dl	84.7 (81.1-91.9)	84.7 (79.3-88.3)	.01
HsCRP, mg/l	2.2 (1.0-5.0)	0.9 (0.4-2.2)	<.001
Total cholesterol, mg/dl	181.8 (162.4-206.9)	177.9 (150.8-197.2)	.02
HDL-cholesterol, mg/dl	54.1 (46.4-61.9)	58.0 (50.3-65.8)	.03
Triglycerides, mg/dl	80.6 (58.5-110.1)	62.9 (47.6-91.2)	<.001

^*^Data are expressed as median (IQR).

*HTP* women with a history of term gestational hypertension or term preeclampsia.

*NTP* women with a history of normotensive term pregnancies.

### Cardiovascular disease risk prediction

#### ***10-year general cardiovascular disease risk prediction by the Framingham risk score***

**Figure 1:**
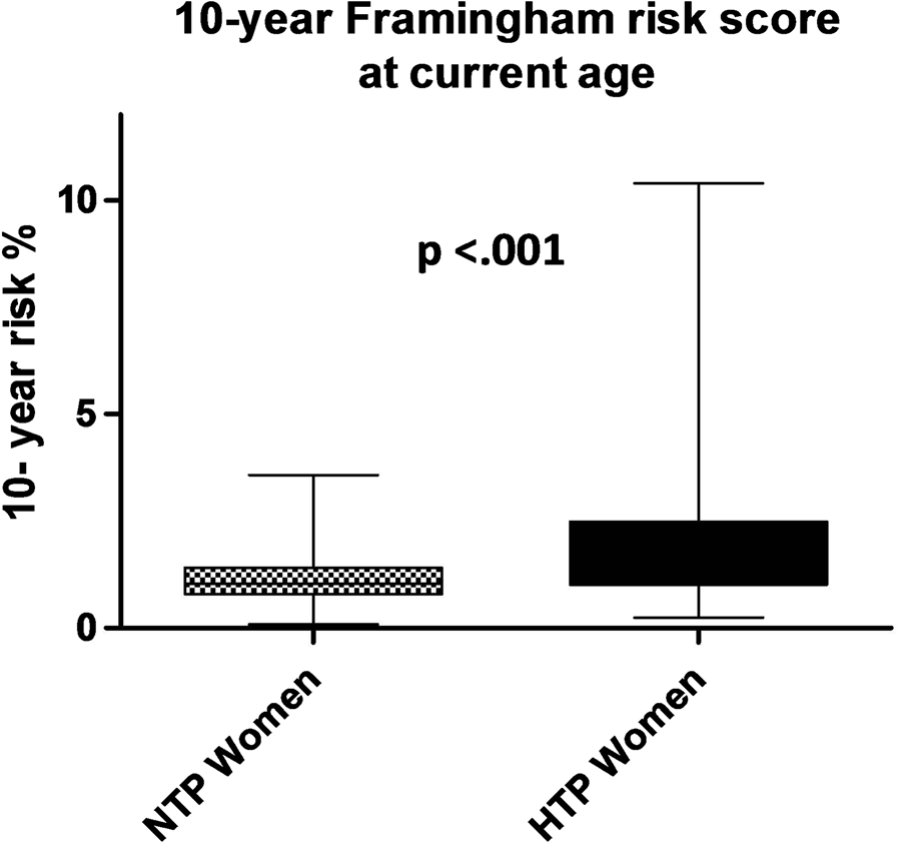
**10 - Year Framingham risk scores (%) of women with a history of gestational hypertension or preeclampsia at term (HTTP women closed bars) and women with a history of uncomplicated normotensive pregnancies (NTP women, dotted bars).** The top and bottom of each box correspond to the 75th percentile and 25th percentile, respectively. The whiskers (t bars) on the top and botttom denote the 90th percentile and 10th percentile, respectively.

Figure 1 illustrates the risk percentages of developing general cardiovascular disease within 10 years in women with a history of term gestational hypertension or term preeclampsia and women with a history of normotensive term pregnancies *at current age*, calculated with the Framingham risk score. At current age, 16 women (5%) with a history of term gestational hypertension or term preeclampsia had a 10-year general cardiovascular disease risk of > 5%, compared with no women (0%) with a history of normotensive pregnancies, p=.02.

**Figure 2:**
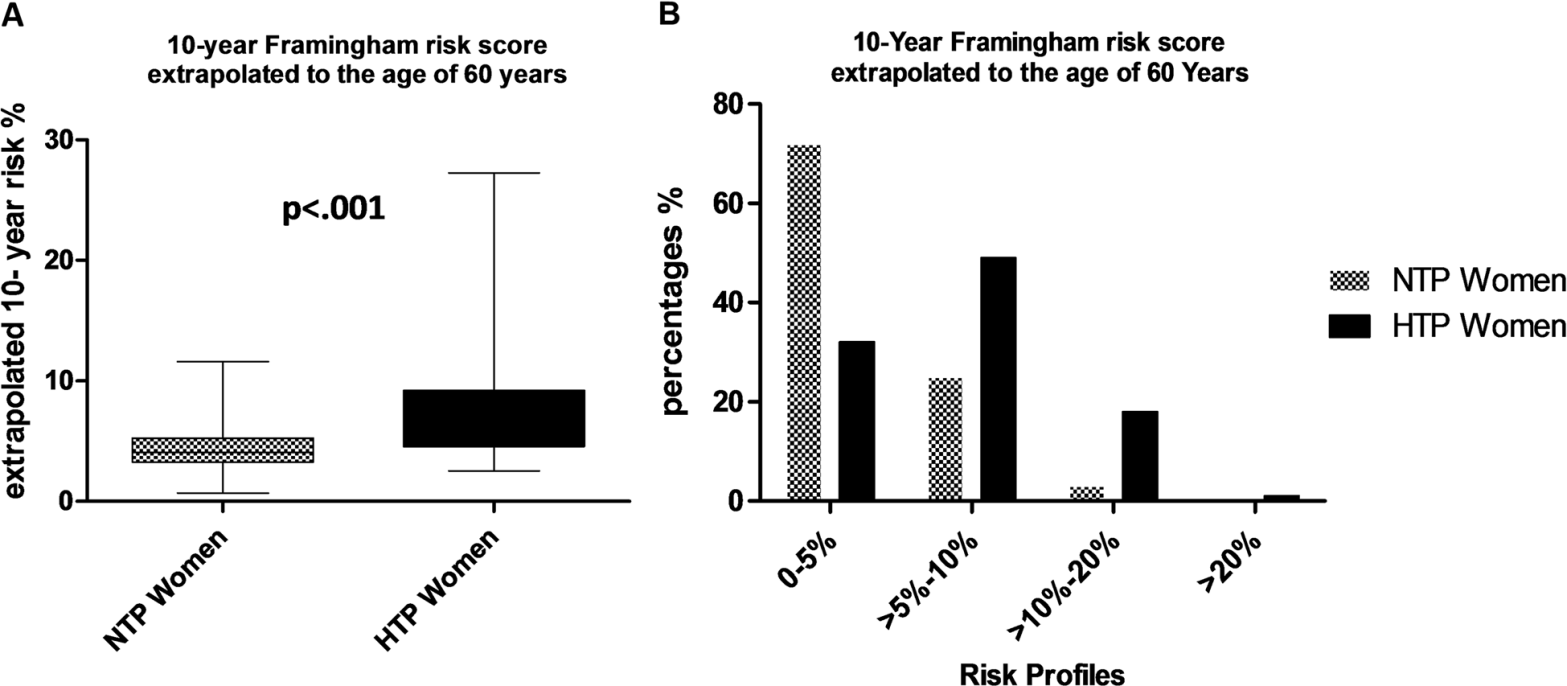
** 10-year Framingham risk score extrapolated to the age of 60 years. A**. 10-Year Framingham risk scores (%) extrapolated to the age of 60 years of women with a history of uncomplicated normotensive pregnancies (NTP women, dotted bars). The top and bottom of each box correspond to the 75th percentile and 25th percentile, respectively. The whiskers (t bars) on the top and botttom denote the 90th percentile and 10th percentile, respectively. **B**. Division into 4 different risk categories (0 - 5%, >5% - 10%>10% - 20% and >20%). 10-Year risk of estimation of overall cardiovascular disease risk according to the Framingham Heart Study algorithm based on the following risk factors, ie. age, smoking, systolic blood pressure, HDL cholesterol, total cholesterol.

Figures 2A and B show the risk percentages of developing general cardiovascular disease within 10 years (Figure 2A) and the different risk categories of the 10-year prediction of developing general cardiovascular disease calculated with the Framingham risk score (Figure 2B) of hypertensive term pregnancy women and normotensive term pregnancy women after extrapolation of their age to 60 years. Poisson regression showed that women with a history of term gestational hypertension or term preeclampsia, compared with women with a history of normotensive term pregnancies, had a 2.5-fold higher extrapolated risk of >5% (p<.001, IRR 2.5 95% CI (1.6 – 3.7)) and even an almost 6-fold higher extrapolated risk of >10% (p=.001, IRR 5.8 95% CI (1.8 - 19)) to suffer from cardiovascular disease within 10-years. After adjustment for parity and current BMI, women with a history of term gestational hypertension or term preeclampsia had still higher extrapolated cardiovascular event risks of >5% (p<.001, IRR 2.3 95% CI (1.5 - 3.5)) and > 10% (p=.01, IRR 5.0 95% CI (1.6 - 16)) compared with women with a history of normotensive term pregnancies.

**Figure 3:**
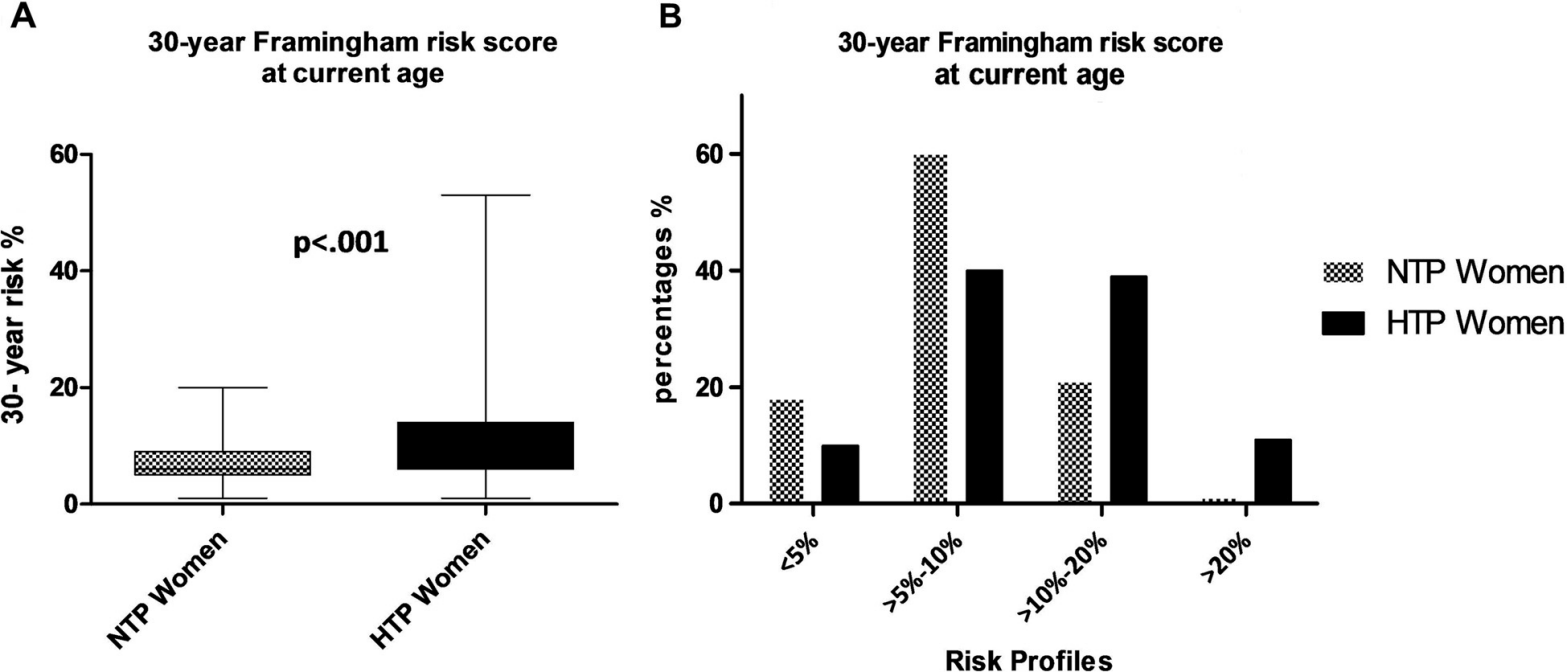
**30-year Framingham risk score at current age. A**. 30 - Year Framingham risk scores (%) at current age of women with a history of gestational hypertension or preeclampsia at term (HTTP women closed bars) and women with a history of uncomplicated normotensive pregnancies (NTP women, dotted bars). The top and bottom of each box correspond to the 75th percentile and 25th percentile, respectively. The whiskers (t bars) on the top and botttom denote the 90th percentile and 10th percentile, respectively. **B**. Division into 4 different risk categories (0 - 5%, >5% - 10%>10% - 20% and >20%). 30 - year risk of estimation of full cardiovascular disease risk by the Framingham Heart Study algorithm based on the following risk factors, ie. age, smoking, systolic blood pressure, HDL cholesterol, total cholesterol, treatment for hypertension and presence of diabetes.

**30-year full cardiovascular disease risk prediction by the Framingham risk score** Figure 3A illustrates the risk percentages of developing overall cardiovascular disease within 30 years in women with a history of term gestational hypertension or term preeclampsia and women with a history of normotensive term pregnancies at current age, calculated with the Framingham risk score. Figure 3B illustrates different risk categories of the 30-year prediction of developing general cardiovascular disease calculated with the Framingham risk score at current age. Poisson regression analysis showed that women with a history of term gestational hypertension or term preeclampsia, compared with women with a history of normotensive term pregnancies had an almost 3-fold higher of >10% (p<.001, IRR 2.7 95% CI (1.6 – 4.5)) to suffer from cardiovascular disease within 30 years, even after adjustment for parity and current BMI (p=.002, IRR 2.4 95% CI (1.4 – 4.1)).

**10-Year fatal cardiovascular disease risk prediction by the SCORE score and 10-Year global cardiovascular disease risk prediction by the Reynolds risk score** Mean (SD) 10-year cardiovascular disease risk predictions and different risk categories calculated by the SCORE score and the Reynolds risk score are shown in Table 4. Women with a history of term gestational hypertension or term preeclampsia had significant higher mean risks calculated by the SCORE score and the Reynolds risk score compared with women with a history of normotensive term pregnancies. Furthermore, women with a history of term gestational hypertension or term preeclampsia were more often represented in higher risk categories.

**Table 4:** Cardiovascular event risk prediction by the SCORE score and Reynolds risk score

**Risk Prediction**	**HTP Cohort (N=300)**	**NTP Cohort (N=94)**	**p value**	**IRR (95% CI)**
***10***-***year fatal CVD risk prediction by the SCORE score (extrapolation to 60 years)***				
Mean (SD), %	1.2 (0.5)	1.1 (0.3)	.02	
Risk category 1%	242 (81%)	85 (90%)		
Risk category 2%	48 (16%)	9 (10%)		
Risk category 3%	9 (3%)	0 (0%)		
Risk category 4%	1 (0.3%)	0 (0%)		
10-year SCORE score risk >1%	58 (19%)	9 (10%)		2.0 (1.0 - 4.1)
***10***-***year global CVD risk prediction by Reynolds risk score (extrapolation to 60 years)***				
Mean (SD), %	2.8 (2.1)	1.6 (1.1)	.001	
Risk category 1%	87 (29%)	60 (64%)		
Risk category >1-5%	168 (56%)	30 (32%)		
Risk category >5-10%	39 (13%)	4 (4%)		
Risk category >10%	6 (2%)	0 (0%)		
10-year Reynolds risk score risk > 5%	45 (15%)	4 (4%)		4.0 (1.0 - 17)

*IRR* incidence risk ratio.

*CI* confidence interval.

*NTP* women with a history of normotensive term pregnancies.

*HTP* women with a history of term gestational hypertension or term preeclampsia.

**Sub-analyses of the hypertensive term pregnancy cohort** We divided the hypertensive term pregnancy cohort (n=300) in a primiparous subgroup (n=134, 44%) and a multiparous subgroup (n=166, 56%) 2.5 years postpartum. Furthermore, we subanalysed women with a history of gestational hypertension at term (n=225, 75%) and women with a history of preeclampsia at term (n=75, 25%) and women with (n=93, 31%) or without (n=207, 69%) severe gestational hypertension or severe preeclampsia during their index pregnancy. No significant differences were found between the subgroups in the prevalence of hypertension 2.5 years postpartum, biochemical cardiovascular risk factors 2.5 years postpartum and in the estimated 10- and 30 year Framingham cardiovascular event risks.

## Discussion

In this longitudinal follow-up study, we found that women with a history of term gestational hypertension or term preeclampsia have an increased 10-year cardiovascular disease risk at current age and after extrapolating the age to 60 years at least two years postpartum. Furthermore, an increased 30-year cardiovascular risk at current age was found in women with a history of term gestational hypertension or term preeclampsia.

### Risk prediction models

Screening and treatment of cardiovascular disease should target women at high risk rather than at women with a single elevated risk factor [12]. Many risk prediction models have been developed the last three decades to estimate individual cardiovascular risk. The Framingham Risk Score was the most influential multivariate risk predictor of developing cardiovascular disease in the future and this algorithm has been most often compared by other studies [15,17,18,27]. The Reynolds Risk Score was developed in women adding CRP and parental history of early myocardial infarction before the age of 60 years as independent risk variables in a female cohort [16] and reclassified 40%-50% of women who were predicted by the Framingham risk score [17] to be at intermediate risk into higher- or lower risk categories. The SCORE equation has been validated for the European population and has been recommended by the Third Joint European Task Force on cardiovascular prevention in Europe [11]. This risk score focuses at fatal total cardiovascular risk rather than at fatal coronary heart disease. Other prediction models were mostly designed and developed for men and therefore not considered for this study. The QRISK score was developed using a population- based clinical research database in the UK incorporating ethnicity, deprivation and other clinical conditions in the algorithm [13,14], resulting in more accurate quantification of risks in south Asian women compared to the Framingham risk score. However, participants in our study were predominantly Caucasian women and adding ethnicity to the risk estimate was considered to have no additional value for risk estimates. Therefore, we did not use the QRISK score for our study. In 2012, Siontis et al. undertook a meta-analysis to compare established risk prediction models for cardiovascular disease [12]. They did not reach robust conclusions about the best risk prediction model or the ranking of performance of different models. An important question rises: which algorithm or model is best suited for women with a history of hypertensive pregnancy disorders at term to accurately estimate their future cardiovascular risk? Unfortunately, we can not answer this question with our present study results.

All four algorithms showed a significantly higher risk in hypertensive term pregnancy women. This is understandable, because the four scores are mostly based on equivalent cardiovascular parameters and these parameters were significantly higher in the hypertensive term pregnancy cohort compared with the normotensive term pregnancy cohort. However, the definitions and cardiovascular endpoints between the four risk prediction models differ. Only the Framingham Heart Study provides a 30-year risk prediction model. This might be the most useful prediction model for our relatively young study cohort, as age remains the most important parameter in cardiovascular risk prediction models and extrapolation is not necessary in this model for our relatively young cohort. Furthermore, the Framingham 30-year risk score focuses at cardiovascular morbidity and mortality rather than at cardiovascular mortality alone (SCORE score), which seems important in young women. Morbidity caused by non fatal cardiovascular events is not only disabling for young women, but it is also the major economic burden for the health care system and society. Young women might benefit from early screening and prevention and it might be cost-effective. However, we have to keep in mind that the longer the prediction period, the less accurate the prediction will be for an individual, as the nature of the design does not account for individual changes in risk factor levels that could have taken place during the course of follow-up [15].

### Comparison with other studies

Three other studies [29,31,32] previously assessed cardiovascular risk scores in women with a history of hypertensive pregnancy disorders. Fraser et al. [31] reported significant higher mean Framingham 10-year cardiovascular risks of 4.6% (0.15) in women with a history of gestational hypertension, 5.1% (0.41) in women with a history of preeclampsia compared with 3.6% (0.06) in women with a history without hypertensive pregnancy disorders. These risks were higher compared with the 10-year cardiovascular risks (at current age) in our study in women with a history of hypertensive pregnancy disorders. This might be explained by two differences.

First, Fraser et al. included not only women with a history of hypertensive pregnancy disorders at term but also women with a history of preterm hypertensive pregnancy disorders, which are known for their higher risk of cardiovascular disease later. Second, the follow-up period of 18 years by Fraser et al. was longer compared with our follow-up period of 2.5 years, which resulted in a significant age difference between the two studies and, per definition, in lower estimation of cardiovascular risk in our study. Smith et al. [29,32] reported comparable 10-year cardiovascular event risks as our study. However, they included women with a history of both preterm and term preeclampsia and they had a shorter follow-up period of 1 year.

Mongraw-Chaffin et al. [33] prospectively investigated the contribution of hypertensive pregnancy complications on the risk of cardiovascular disease death. They found that women with a history of preeclampsia with onset after 34 weeks’ gestation, after a follow-up period of 30 years and with a median age of 56 years, had a cumulative cardiovascular disease death survival of 98.3% and women without a history of preeclampsia had a cardiovascular death survival of 99.3%. We calculated a mean 10-year fatal cardiovascular disease risk of 1.2% (0.5) in our hypertensive term pregnancy cohort after extrapolating the age of each participant to 60 years. This is a slightly lower risk compared with the 1.7% cardiovascular death risk published by Mograw-Chaffin et al. An explanation might be that Mongraw-Chaffin studied women with preeclampsia who delivered after 34 weeks’ gestation, while we included women with both gestational hypertension and preeclampsia after 36 weeks’ gestation. These differences in both gestational age and hypertensive disorder might explain the small discrepancy between the risks. A second explanation might be that hypertensive pregnancy disorders are independent risk factors for cardiovascular disease later in life and as a consequence current cardiovascular risk prediction models underestimate women’s cardiovascular disease risks, as obstetric history is not included as a variable in the models. Further large prospective studies have to evaluate whether hypertensive pregnancy disorders have to be included as an independent variable in cardiovascular risk prediction models for women to improve their assessment of reliable cardiovascular risk estimation for the long term CVD outcome.

### Strengths and limitations

The major strength of this longitudinal follow-up study is that we used a unique prospective cohort from a randomized controlled trial, which consisted of women with a history of term gestational hypertension or term preeclampsia. Hypertensive pregnancy disorders at term are common disorders and therefore of great interest for health care providers. These disorders may be used as a discriminating test whether or not the clinician has to screen for cardiovascular risk factors and calculate subsequent individual cardiovascular risk.

This study has also potential limitations. First, due to the young age of our study participants, 10-year overall cardiovascular disease risk in our cohort was < 5% in all normotensive term pregnancy women and in 95% of hypertensive term pregnancy women in the Framingham Risk Score. For this reason we extrapolated the risk to an age of 60 years. Between current age and the age of 60 years, individual cardiovascular risk factors might change over time. In our study, risk factors were determined at a relatively young age and extrapolation of the age does not account for possible changes in risk factors over time. However, our study method seems an appropriate method considering that both charts of 10 - year prediction after extrapolation to 60 years and charts of 30-year prediction at current age show similar effects.

Second, we performed a cohort study, in which pregnancy data of women with a history of gestational hypertension or preeclampsia were collected at baseline while index pregnancy data of normotensive term pregnancy women were reviewed in detail from medical records at the time of inclusion in the study. However, the information on pregnancy data addressed in this manuscript was nearly all complete in the medical charts.

Third, due to refusal to participate in the follow-up study, pregnant and lactating women at the time of follow-up, and women who were lost to follow-up, we were not able to include the total of 342 women with a history of gestational hypertension or term preeclampsia as described in our power analysis.

Finally, all normotensive term pregnancy women had one or more uncomplicated pregnancies, which might have resulted in a “cardiovascular healthier” cohort compared with a population based cohort and subsequently this relative healthy cohort might have resulted in overestimation of the effect in women with a history of term gestational hypertension or term preeclampsia. However, the mean 10-year cardiovascular disease risk in the Framingham risk study in women of 60 years was 6.4%. The mean extrapolated 10-year cardiovascular disease risk of women with a history of term gestational hypertension or term preeclampsia was higher, namely 7.2%. Assuming that levels of cardiovascular risk factors in women with a history of term gestational hypertension or term preeclampsia, without intervention or prevention, will worsen over time until the age of 60, the real estimated 10-year cardiovascular risk at the age of 60 years of women with a history of term gestational hypertension or term preeclampsia may be even higher than our reported 7.2%. Thus, even without comparison with women with a history of normotensive term pregnancies, a mean 10-year cardiovascular event risk of 7% is of interest for physicians, as it is a higher risk compared with the reported risk of the population based cohort of the Framingham Heart Study.

## Conclusions

In conclusion, women with a history of gestational hypertension or preeclampsia at term have higher (extrapolated) 10-year cardiovascular event risks and 30-year cardiovascular event risks compared with women with a history of uncomplicated pregnancies. Our study results strongly suggest that women with a history of hypertensive pregnancy disorders *at term* may be offered screening and counseling for cardiovascular risk factors after their pregnancy to calculate and estimate cardiovascular event risks.

### Details of ethics approval

The HyRAS and HYPITAT studies were approved both primarily for all participating hospitals in the Netherlands by the medical ethics committee of Leiden University Medical Center (HYPITATP04.210) and locally by the hospital board of the participating hospitals. The clinical trial registration number of the HYPITAT trial is: ISRCTN08132825.

## Abbreviations

HTP: Hypertensive term pregnancy; NTP: Normotensive term pregnancy; IRR: Incidence risk ratio; CI: Confidence interval; SCORE: Systematic coronary risk evaluation; HYPITAT: Hypertension and Pre-eclampsia Intervention Trial at Term; hsCRP: High sensitive C-reactive protein; BMI: Body mass index.

## Competing interests

The authors declare that they have no competing interests.

## Authors’ contributions

WH, JT, AF, JP, MP, KB, MP, BM, and CG designed the study. WH, MVP, KB, JP, MP, BM and CG participated in acquisition of data. WH, DG, JT, BM, and CG analyzed and interpretated the data. WH, DG, JT, BM and CG drafted the manuscript. All authors read and approved the final manuscript.

## Pre-publication history

The pre-publication history for this paper can be accessed here:

http://www.biomedcentral.com/1471-2393/13/126/prepub
